# Utilization of cell-free DNA metagenomic analysis for early detection and microbial identification in prosthetic joint infections: a prospective cohort study in Korea

**DOI:** 10.3389/fcimb.2025.1663857

**Published:** 2025-11-10

**Authors:** Jung Ah Lee, Dongju Won, Eun Hwa Lee, Seung-Tae Lee, Kwan Kyu Park, Saeam Shin, Su Jin Jeong

**Affiliations:** 1 Division of Infectious Diseases, Department of Internal Medicine and AIDS Research Institute, Severance Hospital, Yonsei University College of Medicine, Seoul, Republic of Korea; 2 Department of Laboratory Medicine, Severance Hospital, Yonsei University College of Medicine, Seoul, Republic of Korea; 3 Division of Infectious Diseases, Department of Internal Medicine, Gangnam Severance Hospital, Yonsei University College of Medicine, Seoul, Republic of Korea; 4 Department of Orthopedic Surgery, Severance Hospital, Yonsei University College of Medicine, Seoul, Republic of Korea

**Keywords:** cell-free deoxyribonucleic acid, metagenomics, prosthetic joint infection, synovial fluid, diagnostics

## Abstract

**Background:**

Prosthetic joint infection (PJI) is a severe complication of hip or knee arthroplasty, often necessitating invasive intervention and posing a high risk of adverse outcomes. Early diagnosis and tailored antibiotic therapy are critical for the effective management of PJI. This study evaluated the utility of cell-free deoxyribonucleic acid (cfDNA) extracted from synovial fluid to diagnose PJI and identify the causative pathogens.

**Methods:**

This prospective, single-center study included a PJI group consisting of patients with confirmed infections based on the European Bone and Joint Infection Society criteria and a non-PJI group comprising patients without suspected PJIs who underwent joint surgery or aspiration. Synovial fluid samples were collected from all patients, and various culture methods, including conventional synovial fluid, sonication, and tissue and blood cultures, were applied along with cfDNA analysis.

**Results:**

A total of 35 patients were included, with 20 diagnosed with PJI and 15 classified as non-PJI. The median cfDNA concentration in synovial fluid was significantly higher in the PJI group (4.560 ng/μl, interquartile range (IQR) [3.320–6.348]) compared with the non-PJI group (0.028 ng/μl, IQR [0.009–0.273]) (*p* < 0.001). The Youden index identified a cfDNA concentration ≥ 1.59 ng/μl as strong likelihood of PJI. Culture positivity rates in the PJI group were as follows: synovial culture (10/20, 50.0%), sonication culture (8/9, 88.9%), tissue culture (2/8, 25.0%), and blood culture (2/12, 16.7%). The bacterial detection rate of cfDNA was 65.0% (13/20).

**Conclusion:**

cfDNA concentration was significantly higher in the PJI group, with synovial cultures showing substantial agreement. Additionally, cfDNA sequencing detected pathogens in patients who had received prior antibiotic therapy and identified multiple pathogens in polymicrobial infections. These findings highlight cfDNA analysis as a valuable diagnostic tool for PJI, with the potential to enhance current diagnostic approaches.

## 1 Introduction

Prosthetic joint infection (PJI) is a severe complication of knee and hip arthroplasty, and a leading cause of revision surgery (De Ab Cobra et al., 2022; Khan et al., 2016; Koh et al., 2017). Early detection and precise diagnosis are critical, as PJI impairs function and often necessitates invasive treatment with a high risk of adverse events. However, no universally accepted definition exists for PJIs in clinical practice (Mcnally et al., 2021). This lack of consensus stems from factors including the condition’s complexity, geographical variations in clinical practice, reliance on expensive diagnostic tests, and differing opinions on test accuracy (Villa et al., 2020). Laboratory markers, such as white blood cell counts, polymorphonuclear percentage, C-reactive protein, and erythrocyte sedimentation rate, have been considered highly specific; however, recent studies have shown that cut-off values can vary based on the causative organism (Kheir et al., 2018). Serum markers such as leukocyte esterase, interleukin (IL)-6, IL-8, and alpha-defensin show good diagnostic utility but are prone to false-positive conditions such as metallosis, gout, and other inflammatory diseases (Lee et al., 2017; Saleh et al., 2017; Mcnally et al., 2021). Additionally, synovial culture remains necessary to identify causative microorganisms (Carli et al., 2019). Consequently, diagnosing PJI requires an integrated approach that incorporates clinical assessment, serum and synovial fluid biomarkers, histopathological examination, and microbiological culture (Kheir et al., 2018; De Ab Cobra et al., 2022; Mcnally et al., 2021).

Cell-free deoxyribonucleic acid (cfDNA) comprises extracellular DNA fragments detectable in nearly all biological fluids, including blood, cerebrospinal fluid, urine, and synovial fluid (Chan et al., 2003; De Ab Cobra et al., 2022; Peng et al., 2017). Circulating cfDNA originates from decomposing host cells and microbial invaders that release nucleic acids into body fluids (Jahr et al., 2001). Conventional culture-based methods have limitations, including prolonged turnaround times and difficulty in identifying non-culturable pathogens (Han et al., 2020). Consequently, cfDNA-based pathogen detection is beneficial for identifying various pathogens in PJI and optimizing antibiotic selection. The study evaluated the potential of synovial cfDNA metagenomic analysis for PJI detection and microbial identification, with implications for improving PJI diagnosis and treatment following joint surgery.

## 2 Materials and methods

### 2.1 Study design

**Figure 1:**
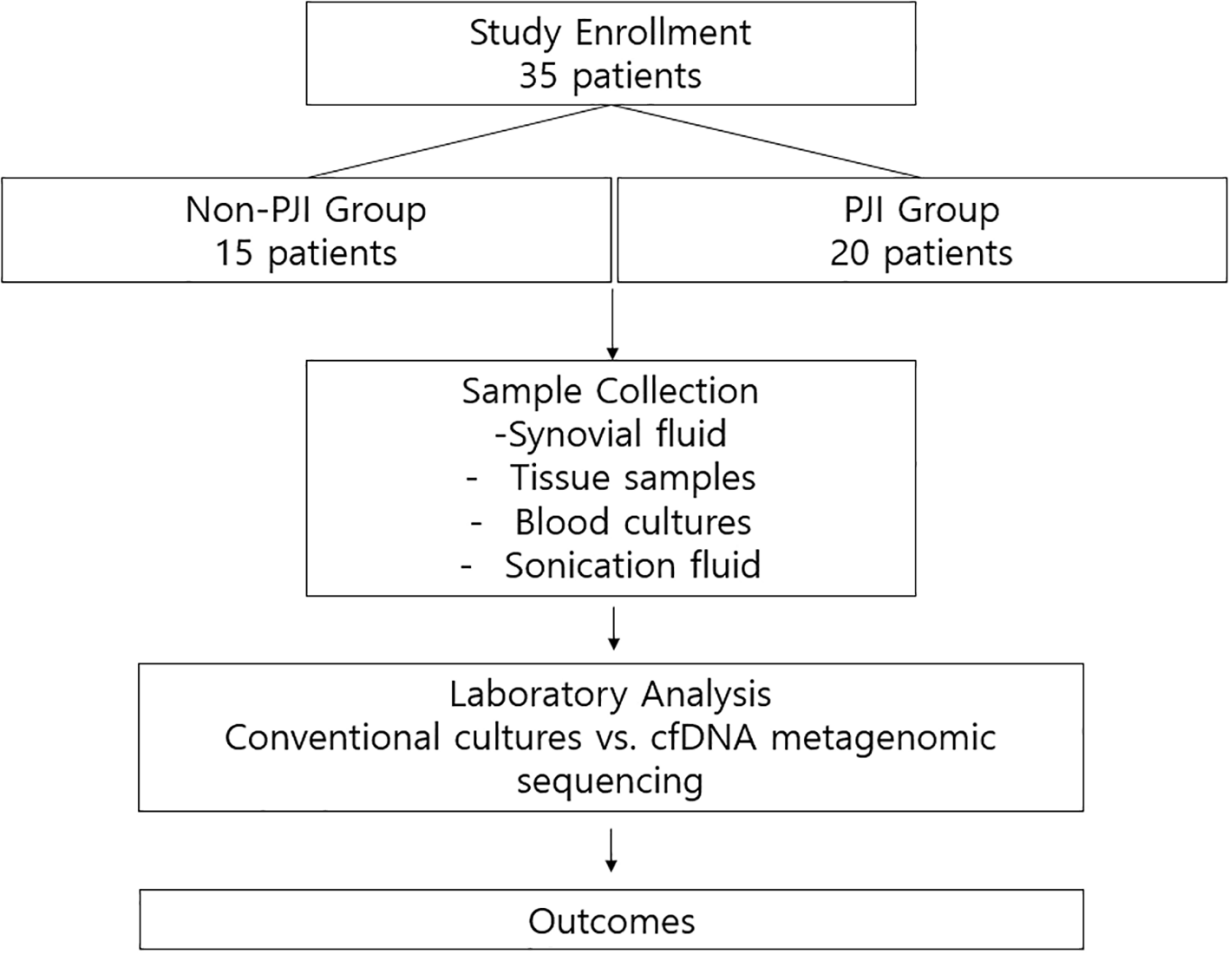
Study design and workflow.

We conducted a single-center prospective diagnostic cohort study at Severance Hospital, a 2400-bed tertiary referral center in Seoul, Republic of Korea. Between June 2020 and September 2023, 35 patients were enrolled, including 20 with and 15 without PJI. This study was approved by the Institutional Review Board of Severance Hospital (No. 4-2019-1060), and written informed consent was obtained from all participants. The overall study design is illustrated in **Figure 1**.

Patients aged ≥ 18 years were eligible for inclusion. The PJI group included patients with confirmed infections based on the definition established by the European Bone and Joint Infection Society (Mcnally et al., 2021). The non-PJI group, which served as a negative control, comprised patients without suspected PJIs who underwent joint surgery or aspiration. Patients aged < 18 years and those who declined participation were excluded.

### 2.2 Sample collection

Synovial fluid was aspirated from all participants upon admission using 18-gauge syringes under sterile conditions. The samples were placed into either plain tubes or transport media, then immediately sent to the microbiology laboratory for analysis. A 2 mL sample was sent for routine fluid analysis, including white blood cell and differential counts, protein and glucose levels, bacterial culture, and cfDNA analysis (Baek et al., 2023).

For microbiological culture, synovial fluid (1 mL) was directly inoculated onto blood agar (BA, Asan Pharmaceutical Co., Ltd., Cat# 29011) and chocolate agar (Choco, Asan Pharmaceutical Co., Ltd., Cat# 29071) plates. Tissue specimens collected during surgery (at least three fragments per case) were inoculated onto blood agar (Cat# 29011), MacConkey agar (MAC, Cat# 29022), thioglycolate broth (Thio, Cat# 28767), and phenylethyl alcohol agar (PE, Cat# 29092), all from Asan Pharmaceutical Co., Ltd. For patients who underwent prosthetic joint removal, sonication fluid was obtained from the explanted prosthesis and inoculated onto blood agar (Cat# 29011), chocolate agar (Cat# 29071), thioglycolate broth (Cat# 28767), and PE agar (Cat# 29092), also from Asan Pharmaceutical Co., Ltd. Aerobic cultures were incubated at 35 °C in 5% CO_2_ for five days, while anaerobic cultures were incubated in an anaerobic chamber at 35 °C for seven days. A culture result was defined as positive if the same microorganism was isolated from at least two separate specimens or aliquots, in accordance with international consensus definitions.

Blood cultures were additionally performed for patients with systemic symptoms suggestive of sepsis (e.g., fever) and incubated for seven days. Tissue samples were also submitted for pathological examination at the discretion of the surgeon.

### 2.3 Data collection

Collected data included age; sex; body mass index; Charlson comorbidity score; steroid and immunosuppressant usage; joint-related comorbidities such as rheumatoid arthritis, osteoarthritis, and gout; location and surgery type; and PJI-related symptoms such as fever, swelling, local tenderness, neurologic deficit, pain, and pus discharge. Admission history, antibiotic use, in-hospital mortality, and recurrence were recorded. Baseline laboratory results, including complete blood count, C-reactive protein, erythrocyte sedimentation rate, blood urea nitrogen, creatinine, albumin levels, and synovial fluid analysis results, were recorded at diagnosis.

### 2.4 Sample preparation and sequencing

Synovial fluid samples were centrifuged at 1900 × *g* for 10 min. The supernatant (0.3 mL) was diluted in 4 mL of 1X phosphate-buffered saline, and cfDNA was extracted using the MagMAX™ Cell-Free Total Nucleic Acid Isolation Kit (Thermo Fisher Scientific, Waltham, MA, USA), according to the manufacturer’s instructions.

cfDNA size and concentration were measured using an Agilent 4150 Tapestation with cfDNA Screen Tape and Reagents (Agilent Technologies, CA, USA) and a Qubit 4.0 fluorometer (Thermo Fisher Scientific). The contaminated samples were processed for double-sized selection using AMPure XP beads. Library preparation was performed using the Twist MF Library Prep with an amp mix kit (Twist Bioscience, San Francisco, CA, USA). Whole-genome sequencing was conducted, and libraries were paired-end sequenced (2 × 150 bp) on a NovaSeq 6000 System (Illumina, San Diego, CA, USA), with 140 million reads for an output of 20 Gb.

### 2.5 cfDNA metagenomic data analysis

Primary sequencing reads were quality-trimmed using Trimmomatic (version 0.39). Reads that passed through these filters were aligned to the human reference genome (GRCh38) using Bowtie2 version 2.3.4.1. Unaligned reads were subsequently mapped to the Kraken2 microorganism reference database using Kraken2 (version 2.1.1). Pavian, an online-based visualization tool, was used to compare the Kraken2 classifications across multiple samples. Reads with consistent patterns across all samples, presumed to originate from skin flora or the environment, were considered as contaminants.

### 2.6 Statistical analysis

All statistical analyses were conducted using the Statistical Package for Social Sciences version 27 (SPSS, Chicago, IL, USA) and R version 4.4.1 (R Foundation for Statistical Computing, Vienna, Austria). The Mann–Whitney U test was employed to compare continuous variables between the PJI and non-PJI groups, while the Pearson χ² test or Fisher’s exact test was used to compare categorical variables. Continuous variables are expressed as medians with interquartile ranges (IQR), while categorical variables are expressed as numbers and percentages. All tests were two-sided, with statistical significance set at *p* < 0.05. Agreement between synovial culture and synovial cfDNA was assessed using Cohen’s kappa statistic. A receiver operating characteristic curve was used to assess the cfDNA concentration as a diagnostic marker for PJI. The Youden index was used to analyze the specific cutoff points for PJI diagnosis.

## 3 Results

### 3.1 Baseline characteristics

Among the 35 participants, 15 had non-PJI, while 20 had PJI. **Table 1** presents the baseline characteristics of the study participants, including infected and non-infected individuals. Participants with PJI showed infection-related symptoms, including fever (*p* = 0.009), swelling (*p* = 0.010), and local tenderness (*p* = 0.001). Pain was observed in both groups. Surgical management in the PJI group included bearing change (n = 6), debridement (n = 1), prostalac insertion (n = 8), and one-stage exchange arthroplasty (n = 5) (**Table 1**).

**Table 1:** Baseline characteristics.

Variables	Not infected (N = 15)	Infected (N = 20)	*p*-value
Age, years, median [IQR]	75 [68.6–81]	70 [64.3–73.8]	0.166
Male sex, n (%)	5 (33.3)	9 (45.0)	0.727
Body Mass Index, kg/m2, median [IQR]	25.6 [23.4–29.0]	26.0 [23.2–28.3]	0.945
Charlson comorbidity index, median [IQR]	3 [1–5.5]	2 [1–6.5]	0.919
Previous arthritis, n (%)			
Osteoarthritis	11 (73.3)	12 (60.0)	0.644
Rheumatoid arthritis	1 (6.7)	1 (5.0)	>0.999
Gout	0 (0)	1 (5.0)	>0.999
Joint, n (%)			0.189
Knee	8 (53.3)	16 (80.0)	
Hip	7 (46.7)	4 (19.0)	
Previous operation, n (%)			**0.001**
Total knee replacement	6 (40.0)	15 (75.0)	
Total hip replacement	0 (0)	3 (15.0)	
Other operations	0 (0)	2 (10.0)	
Not done	9 (60.0)	0 (0)	
Symptom, n (%)			
Fever	0 (0)	9 (45.0)	**0.009**
Pain	15 (100)	20 (100)	>0.999
Swelling	3 (20.0)	14 (70.0)	**0.010**
Local tenderness	3 (20.0)	16 (80.0)	**0.001**
Pus discharge	0 (0)	3 (15.0)	0.338
Surgical management in PJI			
Bearing change		6 (30.0)	N/A
Debridement		1 (5.0)	N/A
Prostalac insertion		8 (40.0)	N/A
One-stage exchange		5 (25.0)	N/A
Prior treatment, n (%)			
Steroid use within 1 month	3 (20.0)	0 (0)	0.138
Chemotherapy within 1 month	0 (0)	2 (10.0)	0.599
Immunosuppressants within 1 month	2 (13.3)	1 (5.0)	0.794
In-hospital mortality, n (%)	0 (0)	2 (10.0)	0.599

Statistically significant results (p < 0.05) are shown in bold.

### 3.2 Difference in cfDNA concentration between non-PJI and PJI cases

**Figure 2:**
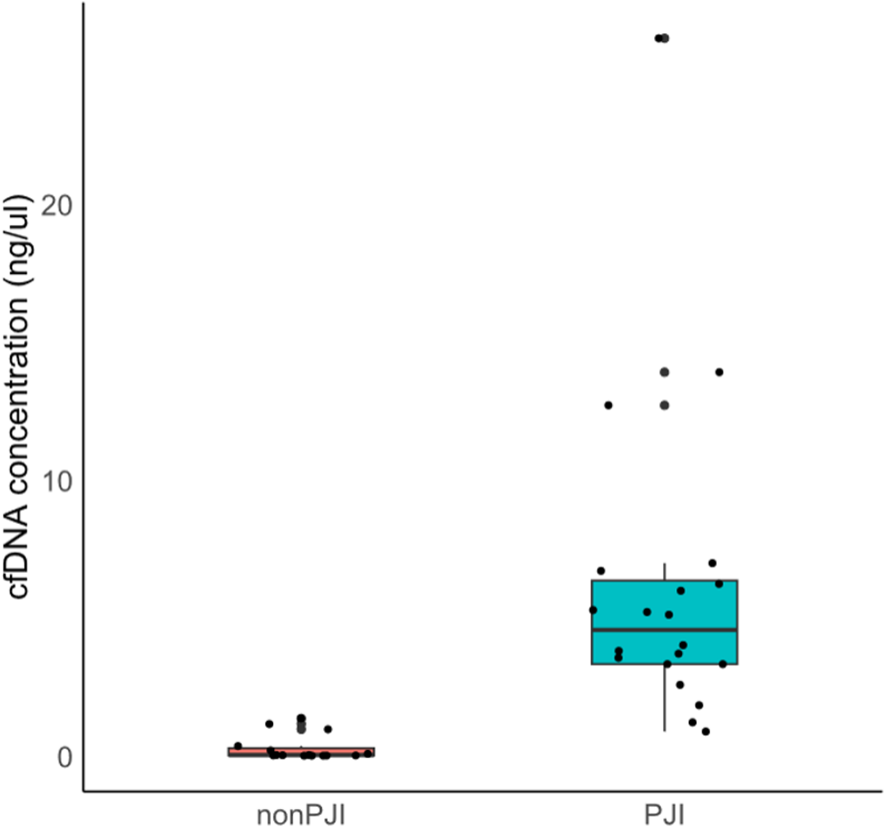
Comparison of synovial fluid cfDNA concentration between PJI and non-PJI groups. cfDNA, cell-free deoxyribonucleic acid; PJI, prosthetic joint infection.

**Figure 3:**
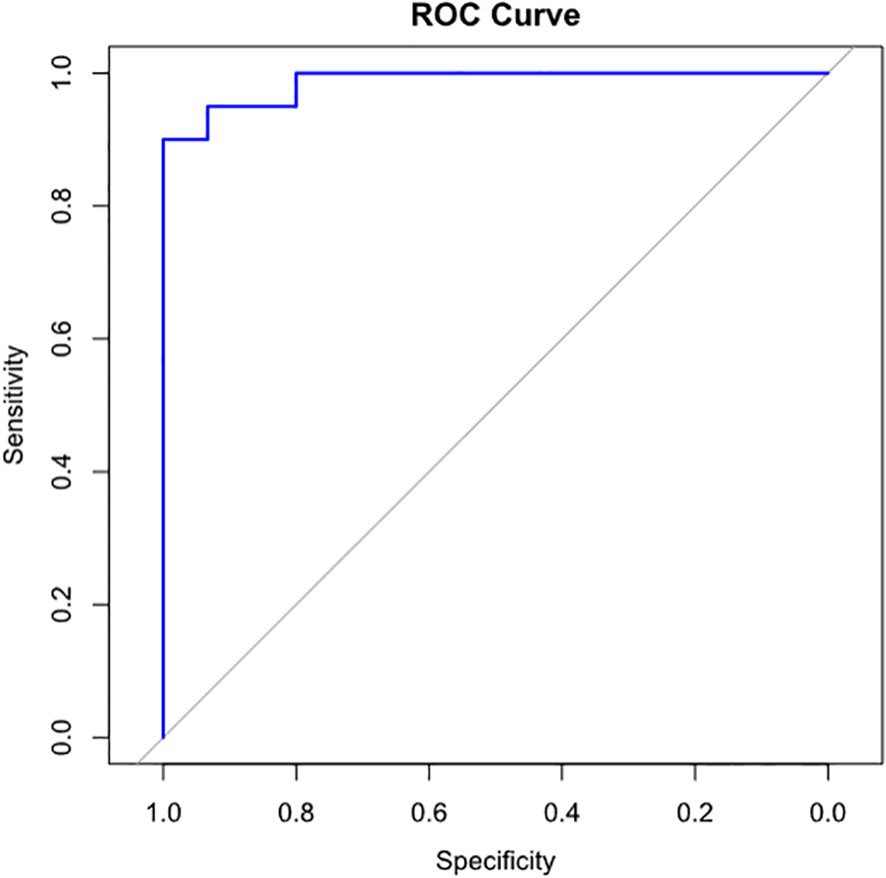
ROC curve for determining the optimal cfDNA concentration cut-off for PJI diagnosis. cfDNA concentration of 1.59 ng/μl or above indicates possibility of PJI. (sensitivity, 0.90; specificity, 1.00), respectively. cfDNA, cell-free deoxyribonucleic acid; PJI, prosthetic joint infection.

The median of cfDNA concentration in the non-PJI group was 0.028 ng/μl [IQR 0.009–0.273], while that in the PJI group was 4.560 ng/μl [IQR 3.320–6.348] (*p* < 0.001). cfDNA concentration in the PJI group was significantly higher than that in the non-PJI group (**Figure 2**). According to the Youden index, a cfDNA concentration of **≥** 1.59 ng/ul indicates a potential PJI diagnosis (**Figure 3**).

### 3.3 Microbiology analysis

In patients with PJI, the culture positivity rates for various methods were as follows: synovial culture yielded positive results in 10/20 cases (50.0%), sonication culture in 8/9 cases (88.9%), tissue culture in 2/8 cases (25.0%), and blood culture in 2/12 cases (16.7%). Bacteria were identified in 15/20 patients (75.0%) using at least one culture method. Bacterial detection via cfDNA metagenomic analysis was observed in 13/20 cases (65.0%), and all cfDNA-positive cases were confirmed as PJI. The most frequently isolated pathogens across all culture techniques and cfDNA analyses included *Staphylococcus* species (eight cases), gram-negative organisms (seven cases), *Streptococcus* species (three cases), and *Enterococcus* species (one case) (**Table 2**).

**Table 2:** Microbiological profiles of the PJI cohort from various culture samples.

Case No.	Synovial culture†	Sonication culture†	Tissue culture†	Blood culture	Other	Synovial cfDNA, Genus level*	Synovial cfDNA, Species level*	Synovial WBC count (/μL)
1	*S. aureus* (Many)	Not done	*S. aureus* (Some)	No growth		*Staphylococcus* (34.2%, 356/1040)		100,000
2	*K. pneumoniae* (Many)	Not done	Not done	No growth		*Klebsiella* (47.6%, 2080/4370)	*K. pneumoniae* (10.9%, 478/4370)	*Uncountable (heavy pus)*
3	*S. dysgalactiae* (Few)	*S. dysgalactiae* (880 CFU)	No growth	No growth		*Streptococcus* (77.4%, 789/1020)	*S. dysgalactiae* (1.1%, 11/1020)	80,000
4	*S. aureus* (Few)	*S. aureus* (880 CFU)	*S. aureus* (Few)	No growth		*Staphylococcus* (16.1%, 257/1600)		49,500
5	*E. coli* (Few)	*E. coli* (40 CFU)	Not done	No growth		*Escherichia* (4.1%, 624/15200)	*E. coli* (2%, 305/15200)	150,000
6	*S. marcescens* (Few)	Not done	Not done	Not done		*Escherichia* (2.3%, 106/4560)	*E. coli* (1.2%, 56/4560)	42,000
7	*S. aureus* (Many)	Not done	Not done	*S. aureus*	*Staphylococcus* sp. (16S rRNA sequencing of tissue)	*Staphylococcus* (84.4%, 13500/16000)		215,000
8	*E. coli* (1 CFU)	Not done	Not done	No growth		*Escherichia* (2.3%, 46/1980) *Streptococcus* (2.1%, 41/1980)	*E. coli* (1.6%, 31/1980)	92,500
9	No growth	*S. aureus* (2800 CFU)	Not done	Not done		Not identified		Not done
10	*S. dysgalactiae* (Some)	*S. dysgalactiae* (>4000 CFU)	No growth	No growth		*Streptococcus* (93.3%, 23500/25200)	*S. dysgalactiae* (2.6%, 650/25200)	*Uncountable*
11	No growth	Not done	Not done	Not done		Not identified		1,955
12	No growth	Not done	No growth	No growth		*Staphylococcus* (66.1%, 1230/1860)	*S. epidermidis* (50.6%, 941/1860)	47,700
13	No growth	Not done	Not done	Not done		Not identified		3,000
14	No growth	Not done	Not done	Not done		Not identified		10,300
15	No growth	Not done	Not done	*Enterobacter* sp.	Synovial aspiration was done after antibiotics use.	*Enterobacter* (36.5%, 1940/5310)		5,100
16	No growth	Not done	Not done	No growth	*S. epidermidis* (1 CFU) (swab culture of op. site in the operation room)	*Staphylococcus* (65.2%, 2760/4230)	*S. epidermidis* (52.5%, 2220/4230)	24,500
17	*E. faecalis* (10 CFU)	*E. faecalis* (>4000 CFU)	Not done	No growth		*Enterococcus* (72.3%, 18000/24900) *Staphylococcus* (15.4%, 3840/24900)	*E. faecalis* (5.7%, 1410/24900) *S. epidermidis* (12.6%, 3130/24900)	Not done
18	No growth	*K. pneumoniae* (2800 CFU)	No growth	Not done		Not identified		Not done
19	No growth	*S. epidermidis* (1200 CFU)	No growth	Not done	*Sphingomonas* sp. (16S rRNA sequencing of tissue)	Not identified		58,320
20	No growth	No growth	No growth	Not done		Not identified		6,000

cfDNA, cell-free deoxyribonucleic acid; WBC, white blood cell; CFU, colony-forming unit; rRNA, ribosomal ribonucleic acid; PJI, prosthetic joint infection.

†The degree of microbial growth is indicated in parentheses.

*Percentage of reads at the genus or species level among all bacterial reads is indicated in parentheses.

### 3.4 Discordance between culture methods and cfDNA analysis

There were seven cases where culture methods and cfDNA analyses yielded discordant results. In Case 6, synovial culture identified *Serratia marcescens*, whereas cfDNA analysis detected *Escherichia coli* as the causative pathogen. In Case 8, both methods identified *Escherichia coli*; however, cfDNA also identified *Streptococcus* species as potential pathogens. In Case 17, both methods identified *Enterococcus faecalis*; however, cfDNA also identified *Streptococcus epidermidis*. In Case 12, synovial culture showed no bacterial growth, whereas cfDNA analysis revealed *Staphylococcus epidermidis*. In Cases 9, 18, and 19, only sonication cultures identified *Staphylococcus aureus*, *Klebsiella pneumoniae*, and *Staphylococcus epidermidis*, respectively. Additionally, in four cases within the PJI cohort, neither culture methods nor cfDNA analysis detected any pathogens.

### 3.5 Comparison of synovial culture and cfDNA analysis

Synovial culture exhibited the highest correlation with cfDNA analysis, with pathogen identification matching in 15/20 cases (75.0%). The agreement between synovial culture and cfDNA results, as measured by Cohen’s kappa statistic, was 0.62, indicating substantial agreement between the two methods.

## 4 Discussion

This study confirmed that cfDNA concentrations in synovial fluid are elevated in patients with PJI and explored the use of cfDNA metagenomic analysis for pathogen identification. PJI is a severe complication following total hip and knee arthroplasty (Ting and Della Valle, 2017), with an estimated economic burden of $60,000–$100,000 per patient and significant morbidity and mortality (Bozic and Ries, 2005; Kurtz et al., 2007; Lavernia et al., 2006). Despite their significant clinical and economic impact, standardized definitions and diagnostic protocols for PJI are lacking (Ting and Della Valle, 2017). Our findings suggest that cfDNA technology may facilitate more rapid and accurate PJI diagnosis. cfDNA-based approaches have been successfully employed in non-invasive prenatal testing, organ transplant rejection monitoring, and liquid biopsy in oncology (Camargo et al., 2019; Bloom et al., 2017; Schütz et al., 2017; De Vlaminck et al., 2014; Allyse and Wick, 2018; Rossi et al., 2018). Recently, this technology has been adapted to diagnose infectious diseases (De Vlaminck et al., 2015; Hong et al., 2018; Lee et al., 2023). In PJI, elevated cfDNA concentrations may enable earlier disease detection, expedite treatment initiation, and improve patient outcomes. This approach could also reduce unnecessary antibiotic use, hospital admissions, and length of hospital stay.

The observed increase in cfDNA concentrations in PJI compared with non-PJI cases was consistent with previous findings. Lögters et al. demonstrated that cfDNA elevation in infections is primarily driven by cell death, particularly through neutrophil extracellular trap (NET) release (Lögters et al., 2009). In a study by Cobra et al., cfDNA concentrations were elevated in the synovial fluid of patients with PJI; however, the source of cfDNA remained unclear, with infecting bacteria proposed as a potential origin (De Ab Cobra et al., 2022). Our study confirmed increased cfDNA concentrations in the synovial fluid of patients with PJI and demonstrated through metagenomic analysis that cfDNA originates from NETosis and bacterial sources.

Rapid diagnosis of PJI and identification of the causative organism are crucial. cfDNA metagenomic analysis detects pathogens at the genus or species level and may provide results faster than traditional culture-based testing (Fung et al., 2017; Hogan et al., 2021). In this study, cfDNA sequencing successfully identified pathogens in three cases where synovial culture failed. Notably, two of these cases involved a history of antibiotic administration within two weeks prior to synovial aspiration. cfDNA sequencing has the advantage of identifying pathogens without being affected by prior antibiotic exposure, a benefit that has been demonstrated in previous studies (Wang et al., 2021). Unlike traditional culture-based methods, which may fail to detect pathogens if antibiotics suppress bacterial growth, cfDNA sequencing detects fragments of microbial DNA present in the bloodstream or infected tissues regardless of the viability of the organism. This advantage makes cfDNA a valuable tool for diagnosing infections, particularly in cases where antibiotics have already been administered.

In comparison with other molecular diagnostic techniques, cfDNA sequencing offers distinct advantages and limitations. Multiplex PCR is rapid but limited to predefined panels, while shotgun metagenomic sequencing (mNGS) provides broader genomic information, including resistance determinants, but is often hampered by high host DNA background and higher costs. cfDNA analysis enriches for extracellular fragments, potentially reducing host interference and enhancing sensitivity, though it cannot yet replace mNGS for resistance profiling. Notably, in two cases, cfDNA sequencing identified multiple microorganisms when synovial culture detected only one, underscoring its potential value in polymicrobial infections.

Sonication culture shows good diagnostic value among prior culture methods compared with conventional bacterial culture with higher sensitivity (Portillo et al., 2014; Liu et al., 2017). In our study, sonication culture identified pathogens that cfDNA analysis did not detect in three cases. This finding suggests that bacteria embedded within biofilms on implant devices may be difficult to detect through cfDNA analysis of the synovial fluid. However, sonication culture requires implant removal, limiting its applicability to post-surgical cases.

Antibiotic selection was based on conventional culture methods (synovial or blood), sonication, or tissue culture, as cfDNA results were not available to clinicians during the study period. However, if cfDNA microorganism results are shared with clinicians, treatment using conventional methods may be enhanced, especially in culture-negative cases. Microbial cfDNA results can help reduce the use of unnecessary antibiotics, leading to antibiotic resistance and side effects. Incorporation of cfDNA could enhance antibiotic stewardship (Yu et al., 2021). Compared with conventional cultures, which typically require 3–7 days for results and are relatively inexpensive, cfDNA sequencing has a higher per-test cost but offers results within 24–48 hours and broader pathogen detection. This trade-off appears reasonable, given the potential to reduce unnecessary antibiotic use and hospital stay through earlier diagnosis.

The possibility of contamination from skin commensals and environmental organisms remains a challenge in cfDNA sequencing. Although we attempted to exclude contaminants by identifying consistent patterns across samples, we acknowledge that whole-genome sequencing (WGS) with SNP analysis would more rigorously confirm whether identical strains are detected by both cfDNA and culture. Due to funding limitations, this was not performed in the present study but will be considered in future investigations.

The primary limitations of this study include its small sample size and single-center design, which may limit the generalizability of the findings. Given the etiological heterogeneity of PJI, a larger, multicenter cohort will be required to validate these results and to improve the reliability of cfDNA-based diagnostic approaches. Additionally, the short read length of the sequencing technology complicates the identification of *Staphylococcus* species. Larger, multicenter studies are required to validate these findings and provide more robust evidence on the diagnostic accuracy of culture methods and cfDNA analysis in PJIs. Another practical limitation is that synovial fluid is not always available in patients with suspected PJI, particularly after synovectomy. While we attempted to include other sample types, the limited number of cases precluded meaningful conclusions. Future studies should incorporate additional specimen types to broaden the applicability of cfDNA testing.

In summary, cfDNA concentrations were significantly higher in patients with PJI, and cfDNA sequencing complemented culture by detecting pathogens in antibiotic-exposed and polymicrobial cases. These findings support cfDNA as a valuable adjunctive tool for improving the diagnostic workflow of PJI.

## Data Availability

The datasets presented in this study are publicly available in the Zenodo repository, DOI: https://doi.org/10.5281/zenodo.17489572
